# Towards elimination of mother‐to‐child transmission of HIV in Rwanda: a nested case‐control study of risk factors for transmission

**DOI:** 10.1186/s12884-021-03806-5

**Published:** 2021-04-28

**Authors:** Eric Remera, Placidie Mugwaneza, Frédérique Chammartin, Augustin Mulindabigwi, Gentille Musengimana, Jamie I. Forrest, Fabian Mwanyumba, Ng’oma Kondwani, Jeanine U. Condo, David J. Riedel, Edward J. Mills, Sabin Nsanzimana, Heiner C. Bucher

**Affiliations:** 1 Institute of HIV Disease Prevention and Control, Rwanda Biomedical Centre, Kigali, Rwanda; 2 Basel Institute for Clinical Epidemiology and Biostatistics, Department of Clinical Research, University Hospital Basel, Basel, Switzerland; 3 University of Basel, Basel, Switzerland; 4 Swiss Tropical and Public Health Institute, Basel, Switzerland; 5 School of Population and Public Health, University of British Columbia, Vancouver, Canada; 6 United Nations International Children’s Emergency Fund, Kampala, Uganda; 7 United Nations International Children’s Emergency Fund, Kigali, Rwanda; 8 School of Public Health, College of Medicine & Health Sciences, University of Rwanda, Kigali, Rwanda; 9 Tulane University, New Orleans, USA; 10 Institute of Human Virology, University of Maryland School of Medicine, Baltimore, USA; 11 Department of Health Research Methods, Evidence, and Impact, McMaster University, Hamilton, Canada

**Keywords:** Mother‐to‐child transmission, HIV, Rwanda

## Abstract

**Background:**

Mother-to-child HIV transmission (MTCT) has substantially declined since the scale-up of prevention programs around the world, including Rwanda. To achieve full elimination of MTCT, it is important to understand the risk factors associated with residual HIV transmission, defined as MTCT at the population-level that still occurs despite universal access to PMTCT.

**Methods:**

We performed a case control study of children born from mothers with HIV with known vital status at 18 months from birth, who were followed in three national cohorts between October and December 2013, 2014, and 2015 in Rwanda. Children with HIV were matched in a ratio of 1:2 with HIV-uninfected children and a conditional logistic regression model was used to investigate risk factors for MTCT.

**Results:**

In total, 84 children with HIV were identified and matched with 164 non-infected children. The median age of mothers from both groups was 29 years (interquartile range (IQR): 24–33). Of these mothers, 126 (51.4 %) initiated antiretroviral therapy (ART) before their pregnancy on record. In a multivariable regression analysis, initiation of ART in the third trimester (Adjusted Odds Ratio [aOR]: 9.25; 95 % Confidence Interval [95 % CI]: 2.12–40.38) and during labour or post-partum (aOR: 8.87; 95 % CI: 1.92–40.88), compared to initiation of ART before pregnancy, increased the risk of MTCT. Similarly, offspring of single mothers (aOR: 7.15; 95 % CI: 1.15–44.21), and absence of postpartum neonatal ART prophylaxis (aOR: 7.26; 95 % CI: 1.66–31.59) were factors significantly associated with MTCT.

**Conclusions:**

Late ART initiation for PMTCT and lack of postpartum infant prophylaxis are still the most important risk factors to explain MTCT in the era of universal access. Improved early attendance at antenatal care, early ART initiation, and enhancing the continuum of care especially for single mothers is crucial for MTCT elimination in Rwanda.

## Background

In 2018, an estimated 1.7 million children (< 15 years) were living with HIV globally and 160,000 were newly infected [1]. The majority of new infections in children (88.1 %) occur in sub-Saharan Africa by vertical transmission which accounts for over 90 % of all HIV mother-to-child transmission (MTCT) worldwide [1, 2]. In 2009, the Joint United Nations Program on HIV/AIDS (UNAIDS) first called for the virtual elimination of MTCT, aiming to reduce vertical transmission to less than 5 % among breastfeeding women and 2 % or less among non-breastfeeding women [3, 4]. By October 2017, eleven countries were found to have eliminated MTCT; none of them is from sub-Saharan Africa [4].

Rwanda has made remarkable strides in increasing the coverage of health facilities providing prevention of mother-to-child HIV transmission (PMTCT) services. In 2012, it became one of the first African countries to recommend the initiation of lifelong antiretroviral therapy (ART) in all pregnant women regardless of CD4 cell count (known as Option B+) [5]. By June 2019, 98 % of health facilities were offering PMTCT services, 99 % of pregnant women attended at least one antenatal care visit, and 96.9 % of HIV-infected pregnant women received ART for PMTCT. As a consequence, the health facility based MTCT rate has decreased to less than 2 % since 2015 [6, 7].

Despite these impressive advances and outcomes, MTCT still occurs in Rwanda. Little is known about the characteristics of mothers whose children acquire HIV infection in the current era of widespread distribution of HIV services and ART. In this study, we investigate risk factors and characteristics of mothers living with HIV whose children did and did not acquire HIV infection.

## Methods

### Study design

Since 2013, the Rwanda Biomedical Centre (RBC) conducts follow-up of children in PMTCT to monitor changes in mother-to-child HIV transmission. To allow for time trend analysis for the fiscal year that ends in June, every year the cohort comprises children born from October to December who are then followed for a period of 18 months. We performed a case-control study that was nested into three consecutive cohorts of children born to mothers with HIV in Rwanda, where cases consisted of vertically infected children and controls were non-infected children by 18 months after birth.

### Study population and setting

The study population included children born to mothers with HIV between October and December 2013, 2014 and 2015 in health facilities that were providing PMTCT services.

At the end of 18 months’ follow-up (time for weaning off), all children who tested HIV positive, were matched with HIV negative children at a ratio of 1:2 by year of birth and health facility to ensure balance between cases and controls. Controls were selected at random from the same facility. When matches within the same health facility were not possible, we considered a paired match from the geographically closest neighbouring health facility.

### Data source

Data on MTCT was collected in 67 out of 517 health facilities offering PMTCT services where we could identify children who were infected with HIV. In these 67 facilities, we selected the cases and controls and abstracted demographics and clinical and laboratory data (extent of viral load suppression at delivery) of the mother from pregnancy to the end of breastfeeding. We anonymized and keyed all data into the Open Data Kit, a free and open-source software for collecting, managing, and using data in resource-constrained countries [8]. From health facility registers, we collected data on children’s HIV status at 18 months after birth, mothers’ age at delivery, companionship by male partner during antenatal care visit, HIV status of male partner, date of antiretroviral therapy initiation for the mother (before pregnancy, during first, second, third trimester of pregnancy, or during labour), mothers’ marital status (single, married, cohabitating, divorced/separated), mothers’ occupation (employed *versus* not employed), mothers’ parity before the current pregnancy (first born, 1–2 children, 3 or more children), place of delivery (health facility *versus* home), mode of delivery (vaginal delivery *versus* caesarean section), maternal HIV viral load at delivery (defined as suppressed if < 1000 copies/ml, and not suppressed if ≥ 1000 copies/ml, and missing data or non-eligible), retention to treatment during antenatal care and breastfeeding, and post-natal ART prophylaxis for new born (yes/no). Retention to treatment was defined as missed drug pick up during three consecutive months from pregnancy to the end of breastfeeding period. The national guideline recommends having the first viral load test after six months on ART and every 12 months subsequently. Thus, mothers who started ART late were more likely to miss the viral load results at the time of delivery. Neonatal ART prophylaxis was defined according to national guidelines as receiving post-delivery ART prophylaxis until the end of six weeks of breastfeeding [9]. Children’s HIV status was defined as a positive or negative HIV test at 18 months after birth. The national HIV guideline recommends follow-up of all children born to mothers with HIV and mothers diagnosed with HIV during the breastfeeding period with HIV testing at 18 months at latest. The follow-up includes HIV DNA PCR test at 6 to 8 weeks and serological tests at 9 and 18 months. Once a serological test is positive, PCR testing is done for confirmation. The child is considered HIV infected if a positive PCR test result is confirmed at any time point, either 6 weeks, 9 months or 18 months after birth [9].

### Statistical analysis

We provide descriptive statistics for characteristics of mothers having given birth to infected and uninfected children with confirmed HIV status at 18 months after birth. Variables that were statistically significant in the univariate analysis (p-value < 0.05) were considered for the multivariable conditional logistic regression model after testing for collinearity. We report adjusted odds ratios with 95 % confidence intervals. All analyses were conducted using Stata version 15 [10].

### Ethical considerations

 This study was performed in accordance with the declaration of Helsinki; the protocol was approved both by the Rwanda National Ethics Committee (reference number: 305/RNEC/2017) and the National Institute of Statistics of Rwanda (reference number: 0667/2017/10/NISR). The Ministry of Health granted approval to access to health facility data to the principal investigator (ER) for the purpose of the study. No participants were involved directly in the data collection therefore their consent was waived by the Rwanda National Ethics Committee. During data extraction, all personal identifiable information was removed to ensure confidentiality of study participants, and fully anonymous identification numbers were created.

## Results

During the three study periods, mothers with HIV gave birth to 5,798 children. Of them, 5,308 (91.5 %) were live births; 125 (2.2 %) infants died after delivery with unknown HIV status, and 365 (6.3 %) children were lost to follow-up at the end of 18 months of follow-up. We identified 84 children with HIV (cases) and confirmed HIV status at 18 months after birth, translating into a vertical transmission rate of 1.6 %. For the purpose of this analysis, all children with HIV were considered as cases and matched with 164 controls (1:2 ratio) from 5,224 HIV negative children. Due to the limited availability of controls in four health facilities and their neighbourhood, four cases were matched with only one control, yielding 164 controls for 84 cases.

The median age of all mothers from both groups at the time of delivery was 29 years (IQR: 24–33), with 27 years (IQR: 22–32) in the case group’s mothers and 29 years (IQR: 25–34) in the control group’s mothers, respectively. Further, dissimilarities were observed between the cases and controls: companionship by a male partner during any antenatal care was lower among mothers of cases (17, 20.2 %) compared to mothers in the controls group (91, 55.5 %). Likewise, 23 (27.4 %) mothers of cases compared to 105 (64.0 %) mothers in the control group started ART before the current pregnancy. A high proportion of mothers of the cases (42.9 %) versus controls (5.5 %) initiated ART during or after labour. In addition, 70 (83.3 %) mothers of cases compared to 157 (96.7 %) mothers of controls were adherent to treatment, and a lower proportion of cases (57.1 %) received ART prophylaxis after birth compared to controls (93.9 %) [Table 1].

**Table 1:** Characteristics of HIV positive mothers by the child’s HIV status at 18 months’ post delivery

	HIV negative children (*n* = 164)	HIV positive children (*n* = 84)	All children (*n* = 248)
	**n (%)**	**n (%)**	**n (%)**
**Age group of mothers (years)**			
below 24	39 (23.8 %)	29 (34.5 %)	68 (27.4 %)
25–34	93 (56.7 %)	40 (47.6 %)	133 (53.6 %)
above 35	32 (19.5 %)	15 (17.9 %)	47 (18.0 %)
**Viral load suppression**			
suppressed (< 1000 copies/ml)	84 (51.2 %)	24 (28.6 %)	108 (43.6 %)
not suppressed ( > = 1000 copies/ml)	47 (28.7 %)	33 (39.3 %)	80 (32.3 %)
missing	33 (20.1 %)	27 (32.1 %)	60 (24.2 %)
**Mothers' marital status**			
single	25 (15.2 %)	24 (28.6 %)	49 (19.8 %)
married	76 (46.3 %)	19 (22.6 %)	95 (38.3 %)
cohabitating	46 (28.1 %)	32 (38.1 %)	78 (31.4 %)
divorced/separated	17 (10.4 %)	9 (10.7 %)	26 (10.5 %)
**Accompaniment by partner during any antenatal care visit**			
no	60 (36.6 %)	42 (50.0 %)	121 (48.8 %)
yes	90 (54.8 %)	16 (19.1 %)	106 (42.7 %)
not documented	14 (8.6 %)	26 (30.9 %)	19 (16.1 %)
**Parity before the current pregnancy**			
no child	49 (29.9 %)	43 (51.2 %)	92 (37.1 %)
1–2 children	89 (54.3 %)	33 (39.3 %)	122 (49.2 %)
3 + children	26 (15.8 %)	8 (9.5 %)	34 (13.7 %)
**HIV status of the partner**			
HIV positive	76 (46.3 %)	26 (30 %)	102 (41.1 %)
HIV negative	39 (23.8 %)	8 (9.5 %)	47 (19 %)
unknown	49 (29.9 %)	50 (59.5 %)	99 (39.9 %)
**ART initiation**			
before pregnancy	105 (64.0 %)	23 (27.4 %)	128 (51.6 %)
first or second trimester of pregnancy	40 (24.4 %)	10 (11.9 %)	50 (20.2 %)
third trimester of pregnancy	10 (6.1 %)	15 (17.8 %)	25 (10.1 %)
during or after labour	9 (5.5 %)	36 (42.9 %)	45 (18.2 %)
**Place of delivery**			
home	8 (4.9 %)	17 (20.2 %)	25 (10.1 %)
health facility	156 (95.1 %)	67 (79.8 %)	223 (89.9 %)
**Retained in care during pregnancy or breastfeeding period**			
yes	157 (95.7 %)	70 (83.3 %)	227 (91.5 %)
no	7 (4.3 %)	14 (16.7 %)	21 (8.5 %)
**Mode of delivery**			
vaginal delivery	143 (89.4 %)	65 (83.3 %)	208 (87.4 %)
caesarean section	17 (10.6 %)	13 (16.7 %)	30 (12.6 %)
**ART prophylaxis for the child at birth**			
yes	154 (93.9 %)	48 (57.1 %)	202 (81.5 %)
no	10 (6.1 %)	36 (42.9 %)	46 (18.6 %)
**Year of birth of child**			
2013	64 (39.0 %)	34 (40.5 %)	98 (39.5 %)
2014	46 (28.1 %)	23 (27.4 %)	69 (27.8 %)
2015	54 (32.9 %)	27 (32.1 %)	81 (32.7 %)

*ART:* antiretroviral therapy

In adjusted analyses, the odds of MTCT with initiation of ART in the third trimester and during labour or post-partum compared to ART initiation before pregnancy were 7.13 (95 % CI 1.82- 27.908) and 8.97 (95 % CI 2.38–33.69.), respectively. Children from single mothers with HIV were at increased risk of HIV infection (aOR 6.93; 95 % CI 1.53–31.30) compared to non-single mothers. Consultation without companionship by male partner during antenatal care visits was also associated with increased risk for MTCT (aOR 4.48; 95 % CI 1.12–17.82), and the odds of HIV infection for children who missed ART prophylaxis at birth was higher compared to those who did received it (aOR 7.26; 95 % CI: 1.66–31.59) [Table 2].

**Table 2:** Factors associated with mother to child HIV transmission

	Bivariate analysis		Multivariable analysis	
	**OR**	**95 % CI**	**aOR**	**95 % CI**
**Age group of mothers (years)**				
below 24 (ref)	1	**-**		
25–34	0.51	0.25–1.06		
above 35	0.59	0.23–1.58		
**Viral load suppression**				
suppressed (< 1000 copies/ml) (ref)	1	-	1	-
not suppressed ( > = 1000 copies/ml)	2.96*	1.43–6.14	1.95	0.61–6.27
missing	4.17*	1.75–9.91	1.88	0.53–6.70
**Marital status**				
married (ref)	1	-	1	-
single	8.23**	2.92–23.22	6.93*	1.53–31.30
cohabitating	4.33**	1.88–9.99	3.89	0.92–16.43
divorced/separated	2.53	0.89–7.19	1.07	0.21–5.50
**Occupation**				
employed (ref)	1	-		
not employed	1.36	0.61–3.05		
**Parity before the current pregnancy**				
1–2 children (ref)	1	-	1	-
no child	2.75*	1.46–5.15	1.86	0.65–5.35
3 and more	0.74	0 0.29-1.89	1.61	0.45–5.75
**Accompaniment by partner during any antenatal care visit**				
yes (ref)	1	-	1	-
no	4.80**	2.52–9.17	1.93	0.72–5.22
not documented	2.93	0.96–8.87	1.03	0.16–6.62
**Initiation of ART**				
before pregnancy (ref)	1	-	1	-
first or second trimester of pregnancy	1.58	0.64–3.89	2.15	0.68–6.79
third trimester of pregnancy	7.27**	2.40-22.04	7.13**	1.82–27.90
during or after labor	16.43**	6.16–43.75	8.97**	2.38–33.69
**Place of delivery**				
health facility (ref)	1	-	1	-
home	4.43*	1.83–10.75	1.41	0.27–7.46
**ART prophylaxis for the child at birth**				
yes (ref)	1	-	1	-
no	9.54**	4.23–21.51	5.76**	1.62–20.47
**Retained in care during pregnancy or breastfeeding period**				
yes (ref)	1	-	1	-
no	4.17*	1.59–10.96	2.44	0.53–11.24
**Mode of delivery**				
vaginal delivery (ref)	1	-		
caesarean section	1.64	0.73–3.68		
**Year of birth of child**				
2013 (ref)	1	-		
2014	0.93	0.10–8.63		
2015	0.61	0.07–5.24		

*p-value < 0.05, **p-value < 0.001

## Discussion

Rwanda has achieved a low level of facility based MTCT with a rate of less than 2 % for children whose mothers were followed in the PMTCT programme. As the country is on the last mile to reach the elimination of MTCT, we identified key factors that were associated with increased risk of MTCT using a case-control study from a representative cohort of mothers with HIV who had given birth to children in a three-month period of 2013, 2014, and 2015 when ART use was widely available in the country. These results showed that late ART initiation (in the third trimester of pregnancy and during labour or post-partum), being a single mother, lack of male involvement during antenatal care, and missing postpartum neonatal prophylaxis were all independently associated with the risk of MTCT. Although these findings are not novel risks for MTCT, they identify gaps that still remain in the Rwandan national HIV programme response.

Since 2013, Rwanda has initiated the test and treat for pregnant women attending antenatal clinic and, consequently, the country has reached a high level of ART coverage for pregnant women of 97 % in 2019 [7]. The secondary analysis of the Rwanda demographic health survey (RDHS) conducted in 2015, shows that 7.1 % of pregnant women with HIV attend the first antenatal visit in the third trimester of pregnancy and findings of this study show a high risk of MTCT for mothers who started ART in the third trimester of pregnancy and during labour and in those who were not retained in care during pregnancy or post-partum. Similar results were reported in studies conducted in Malawi, Zimbabwe [11, 12]. Even though antenatal care participation was high in this study, early ART uptake is crucial to prevent MTCT and should most preferably be initiated before pregnancy or in the first trimester of pregnancy at latest [13]. With the current coverage of 97 % in Rwanda, most pregnant women are very likely to be on ART and the rate of MTCT in Rwanda is expected to decrease if strategies are put in place to address loss to follow-up and to ensure HIV positive mothers and their exposed infants are retained within the continuum of care. Different studies revealed that non-retention in care among pregnant women may be due to the fear of disclosure, stigma and insufficient social support [11, 14].

Rwanda has observed a high male accompaniment of above 85 % in antenatal care for both mothers with and without HIV [15]. Despite many efforts centred on increasing the male involvement in maternal and child health, results of this analysis showed that only 46 % of mothers with HIV attended ANC accompanied by their male partners, and there was a high risk of vertical HIV transmission for mothers who were not accompanied by their male partners in antenatal care. A study conducted in Kenya highlighted that HIV-infected women were less likely to disclose their status to partners than HIV-uninfected women [16], while other studies suggest the role of male partner participation in antenatal care is critical to ensure infant survival and HIV infection among children born to HIV infected mothers [17–19].

Our findings showed that missing infant postpartum prophylaxis increases the risk of MTCT. These results are comparable with findings from a cross sectional study at 6–10 weeks postpartum in HIV positive mothers in Rwanda who were on ART during pregnancy and breastfeeding to estimate the MTCT [13] and similar results were also reported in retrospective studies conducted in Kenya, highlighting the risk of MTCT for infants missing the post-partum antiretroviral prophylaxis [20, 21].

Further, in this survey young and single mothers who did not receive a full package of ART according to the Rwanda EMTCT protocol had an increased risk of MTCT. Although we did not have information whether their pregnancies were intended or not, other studies conducted in sub-Saharan Africa have reported high transmission rates among non-married mothers, particularly women who did not intend to become pregnant [22, 23]. Adolescent girls and young women (AGYW) represent a vulnerable sub-group in the HIV epidemic. In sub-Saharan Africa, three in five new HIV infections are among females below 20 years of age [24]. Young females appear to be at higher risk of HIV infection than their male peers due to male violence, lack of awareness of HIV and of access to sexual reproductive health care services, lack of education and health care policies which do not sufficiently translate into actions to protect this vulnerable risk group [25]. In Rwanda, different interventions targeting AGYW were introduced in 2018 through the DREAMS program supported by the U.S. President’s Emergency Plan for AIDS Relief with the aim of providing access to education, improving the economic empowerment and increasing the awareness on HIV and sexual reproductive health among AGYW. The DREAMS program is implemented in 10 countries in Sub-Saharan Africa with the goal of reducing HIV incidence by 40 % among AGYW [26].

### Strengths and limitations

Our analysis has both strengths and limitations. This is the first case control study ever conducted at national scale in Rwanda using data from three cohorts from different years. However, given that the study was retrospective, we experienced some limitations: firstly, we could only work with available information recorded in the health registers. Although viral load is important in determining the risk of MTCT, we were not able to collect the viral load at the time of delivery for women who were enrolled on ART in the last trimester of pregnancy, as according to local guidelines the first viral load test is done at six months after ART initiation. Secondly, given that the electronic medical record is not yet used across all the health facilities and services, all information from mothers who had been transferred from another health facility were not available. Lastly, HIV status at 18 months was not known in 8.5 % of children of the target population because they had died or were lost to follow-up, introducing a potential selection bias that could not be avoided.

## Conclusions

Findings from this study indicate a strong need to identify and treat reproductive age women with HIV as soon as possible and enrol them even before conception into antenatal care programs to ensure retention in care and administer prompt postpartum ART prophylaxis to children. Further, there is a need to continue focusing on vulnerable AGYW through programs like DREAMS to empower women and consequently reduce MTCT and to assess the impact of newly initiated interventions targeting AGYW on the reduction of the MTCT. The continued decline of new infections of infants also would reduce the programmatic burden to give space for focused care of those most in need.

## Data Availability

Raw data used in article are available upon reasonable request in writing to the corresponding author (ericremera@gmail.com) and will be deposited in a public repository as soon as we gain permission.

## Abbreviations

HIV: Human Immunodeficiency Virus
MTCT: Mother to Child HIV Transmission
PMTCT: Prevention of Mother to Child HIV Transmission
EMTCT: Elimination of Mother to Child HIV Transmission
aOR: Adjusted Odds Ratio
CI: Confidence Interval
IQR: Interquartile range
ART: Antiretroviral therapy
RBC: Rwanda Biomedical Centre
UNAIDS: Joint United Nations Program on HIV/AIDS
PCR: Polymerase Chain Reaction
RNEC: Rwanda National Ethics Committee
AGYW: Adolescent girls and young women
